# The effects of tannins-containing ground pine bark diet upon nutrient digestion, nitrogen balance, and mineral retention in meat goats

**DOI:** 10.1186/s40104-015-0020-5

**Published:** 2015-06-05

**Authors:** Byeng Ryel Min, Sandra Solaiman, Thomas Terrill, Aina Ramsay, Irene Mueller-Harvey

**Affiliations:** Department of Agricultural and Environmental Sciences, Tuskegee University, Tuskegee, AL USA; Agricultural Research Station, Fort-Valley State University, Fort-Valley, GA USA; School of Agriculture, Policy and Development, University of Reading, P.O. Box 236, Berkshire Reading, RG6 6AT UK

**Keywords:** Digestibility, Goats, Phytochemicals, Tannins

## Abstract

**Background:**

Pine bark is a rich source of phytochemical compounds including tannins, phenolic acids, anthocyanins, and fatty acids. These phytochemicals have potential to significantly impact on animal health and animal production. The goal of this work is to measure the effects of tannins in ground pine bark as a partial feed replacement on feed intake, dietary apparent digestibility, nitrogen balance, and mineral retention in meat goats.

**Results:**

Eighteen Kiko cross goats (initial BW = 31.8 ± 1.49 kg) were randomly assigned to three treatment groups (*n* = 6). Dietary treatments were tested: control (0 % pine bark powder (PB) and 30 % wheat straw (WS)); 15 % PB and 15 % WS, and 30 % PB and 0 % WS. Although dry matter (DM) intake and digestibility were not affected (*P* > 0.10) by feeding PB, neutral detergent fiber (linear; *P* = 0.01), acid detergent fiber (linear; *P* = 0.001) and lignin digestibility (linear; *P* = 0.01) decreased, and crude protein (CP) digestibility tended to decrease (*P* = 0.09) as PB increased in the diet, apparent retention of Ca (*P* = 0.09), P (*P* = 0.03), Mg (*P* = 0.01), Mn (*P* = 0.01), Zn (*P* = 0.01) and Fe (*P* = 0.09) also increased linearly. Nitrogen intake and fecal N excretion were not affected (*P* > 0.05) by addition of PB in the diet, but N balance in the body was quadratically increased (*P* < 0.01) in the 15 % PB diet compared to other diets. This may be due to more rumen escape protein and less excreted N in the urine with the 15 % PB diet. The study showed that a moderate level of tannin-containing pine bark supplementation could improve gastrointestinal nitrogen balance with the aim of improving animal performance.

**Conclusion:**

These results suggest that tannin-containing PB has negative impact on fiber, lignin, and protein digestibility, but positively impacted on N-balance.

## Background

Condensed tannins (CT) are, prevalent in many plants and, may reduce ruminal protein degradation, which can increase intestinal protein flow when provided at moderate doses of 2 to 4 % CT in the dry matter (DM) [1]. However, Barry and Manley [2] reported that digestibility of all nutrients was reduced when sheep were fed high-CT (>5 % CT DM) containing *Lotus pedunculatus*. Commercial Quebracho CT added to a CT-free diet similarly reduced protein digestibility in a dose-dependent manner in ruminants [3]. However, goats are predominantly browsers and able to consume larger amounts of tannin-rich browse than sheep under similar conditions [4] without any signs of toxicity [5]. Recently, Min et al. [6, 7] have described the improvement of animal performance and average daily gain (ADG) in meat goats fed CT-containing ground pine bark (PB; 0, 15, and 30 % PB/kg of DMI) without any detrimental effects. Therefore, the source of CT, as well as its concentration, needs to be considered in studies involving protein degradation and plasma blood metabolisms. The objective of this study was to assess the effects of different levels of CT-containing PB on ruminal digestibility, nitrogen balance, and mineral retention in goats.

## Materials and methods

### Experimental design

Eighteen Kiko-cross meat goats (*Capra hircus*; body weight (BW) = 31.0 ± 1.49 kg) were fed three different levels of CT-containing ground PB (*Pinus taeda* L.) to study the effects of CT in ground PB upon nutrient digestion and metabolism and upon animal performance. The study was conducted at the Caprine Research and Education Center, Tuskegee University, Tuskegee, AL. Goat kids, approximately 5 month of age, were stratified by BW and randomly assigned to the experimental treatments in a completely randomized design with two different periods. In the 2nd period, new animals were randomly allocated to the experimental treatment with the same dietary treatments. A preliminary period of at least 30 days was allowed for the animals to adjust to each ration before 7 days of fecal collection periods.

Animals were examined and drenched with anthelmintic (Cydectin; Moxidectin, Fort Dodge Animal Health, Fort Dodge, Iowa, USA). These goats were housed indoors in metabolism crates and offered a mixed diet (Table 1) with 15 % bermudagrass hay (BGH; *Cynadon Dactylon*). All the diets were weighed before and after being offered to measure DMI. Mixed diets contained different levels of the ground PB replacing ground wheat straw (WS; *Triticum aestivum*). Experimental treatments included: the control diet–0 % PB plus 30 % WS, 15 % PB plus 15 % WS, and 30 % PB plus 0 % WS.

**Table 1:** Ingredients and chemical composition of experimental diets and diet ingredients, i.e. pine bark (PB), wheat straw (WS), and bermudagrass hay (BGH)

	Grain Mix (% PB), %			Ingredient, %			
Item	0	15	30	SEM	PB	WS	BGH
Ingredient of the grain/pine bark mix, % as is							
Ground pine bark	0	15	30	-	-	-	-
Ground wheat straw	30	15	0	-	-	-	-
Corn	20	20	20	-	-	-	-
Soybean meal, 48 % CP	18.5	20	21	-	-	-	-
Soy hulls	4.5	5	4	-	-	-	-
Alfalfa meal	5	3	3	-	-	-	-
Molasses	6	6	6	-	-	-	-
Vitamins and mineral mix^a^	0.5	0.5	0.5	-	-	-	-
Salt	0.5	0.5	0.5	-	-	-	-
NH_4_Cl	0.5	0.5	0.5	-	-	-	-
BGH	15	15	15	-	-	-	-
Chemical composition, % DM (*n* = 3)							
DM	89.7	87.8	87.3	0.77	83.6	83.5	91.4
CP	15.7	16.8	16.1	0.41	1.2	4.1	7.3
ADF	23.7	23.2	23.6	1.42	72.1	49.2	37.3
NDF	35.0	31.8	27.5	1.77	78.6	79.0	69.2
NFC^b^	42.1	42.5	47.1	1.91	17.1	16.7	19.1
Ash	6.4	6.2	5.9	0.31	2.25	2.0	4.84
Lignin	5.9	9.9	12.4	0.85	21.3	8.01	6.29
Ether Extract	2.3	2.6	2.5	0.25	1.65	0.42	1.51
TDN	66.6	64.1	64.4	1.75	36.7	52.0	56.3
NE_m_ (Mcal/kg)	0.31	0.30	0.30	0.01	0.10	0.21	0.54
NE_g_ (Mcal/kg)	0.19	0.17	0.18	0.01	0.10	0.10	0.28
Ca	0.61	0.56	0.53	0.04	0.25	0.17	0.39
P	0.35	0.38	0.37	0.02	0.04	0.08	0.19
Mg	0.23	0.23	0.24	0.01	0.02	0.05	0.24
K	1.19	1.12	1.05	0.03	0.03	0.31	0.99
S	0.21	0.22	0.22	0.09	0.01	0.01	0.20
Na	0.10	0.10	0.08	0.08	0.08	0.04	0.01
Cu, ppm	34.7	25.3	19.7	8.01	1.0	5.0	3.0
Mn, ppm	118.3	108.3	94.3	12.0	30.0	63.0	43.0
Zn, ppm	133.0	142.3	152.0	14.6	11.0	5.0	20.0
Fe, ppm	192.7	203.6	196.6	19.09	384	111	211.3
CT, % DM^c^	0.19	1.63	3.20	0.19	10.3	0.03	0.04

^a^ Guaranteed analysis: calcium, 9.0 %; phosphorus, 8.0 %; salt, 41 %; potassium, 0.10 %; copper, 1750 ppm; selenium, 25 ppm; zinc, 7500 ppm; vitamin A, 308,000 IU/kg; vitamin D, 24,200 IU/kg; vitamin E, 1650 IU/kg

^b^ *NFC* non-fiber carbohydrate. NFC was calculated by difference [100 – (%NDF + %CP + %Fat + Ash)]

^c^ Condensed tannins (CT) are relative to a purified Quebracho condensed tannins standard (on DM basis). *TDN* total digestible nutrient, *DM* dry matter, *CP* crude protein, *NDF* neutral detergent fiber, *ADF* acid detergent fiber, *CT* condensed tannins

An adjustment period of 4 weeks allowed goats to be acclimated to pen living, routine feeding and to adapt to the environment and feed prior to collecting measurements. Animals (*n* = 6) were individually fed at 0900 h and feed offered and refused was monitored for 7 days of total fecal and urine measurements. Animals had access to water and trace mineral salt block ad libitum. Grain mixes containing ground PB/WS were commercially mixed at the local feed mill (Eclectic Feed Mill, 3180 Chana Creek Rd., Eclectic, AL 36024) and were offered daily at 85 % of the total ration, with the remaining 15 % consisting of BGH. Grain mix and long BGH were offered separately by weight basis and refusals were recorded daily. During the adjustment phase, the quantity of diet offered was increased sequentially until refusals reached or exceeded 5 % of that provided. Used as an estimate of ad libitum consumption by each animal, this intake level was maintained during the collection period. Two main dietary sources were used in this study; the first PB contains CT, whilst the second, WS contains little or no CT. PB was used as a source of CT and was substituted for WS in the diet. For this study, WS were chosen as a negative control because initial chemical analysis data (Table 1) showed that neutral detergent fiber (NDF) and non fibrous carbohydrate (NFC) contents were similar to PB. Diets contained different levels of CT-containing ground PB and replaced ground WS. Goats were provided diets that met all animal requirements for growth and gain [8].

The fresh PB was donated by a wood processing company (West Frazer, 2100 Industrial Blvd., P.O. Box 4230, Opelika, AL 36801), and air-dried in a shed before processing. Freshly dried PB and WS were finely (1.5-3 mm) ground (Hammer Mill Model 1250; Lorenz MFG Co., Benson, MN, USA) and incorporated in the grain mix portion of the diets to provide 1.9, 16.3, and 32 g CT/kg DM in 0, 15, and 30 % PB/WS diets, respectively (Table 1). The Tuskegee University Animal Care and Use Committee approved all animal care, handling and sampling procedures used in this study.

### Sample collection and laboratory analysis

During the collection periods, total dietary, fecal and urine samples were collected at 0900 h during 7 days. For laboratory analysis, individual fecal samples were collected daily in a 10-L plastic bucket, weighed and composited for each animal (10 %) and stored at −20 °C for later analysis. Urine, collected daily in 4-L jugs containing 10 mL of 50 % HCl, was weighed and a sample of urine was composited for each animal (10 %) and stored frozen for later analysis. Samples of feed and feces were dried at 55 °C to constant weight in a forced air oven (model 420, NAPCO, Pittsburgh, PA) during 48 h. Dietary and fecal samples were ground in a Thomas-Wiley mill (model 4, Thomas Scientific, Philadelphia, PA) to pass through a 1-mm mesh screen. Daily portions of ground samples were composited for each animal and analyzed for DM, crude protein (CP), NDF, acid detergent fiber (ADF), lignin, ash, non-fiber carbohydrate (NFC), ether extract, total digestible nutrient (TDN), net energy for maintenance (NE_m_), net energy for gain (NE_g_), and minerals according to the methods described by AOAC [9]. NFC was calculated by difference [100 – (%NDF + %CP + %Fat + Ash)]. Nitrogen for diet and fecal samples was determined using Kjeldahl N, and CP was calculated by multiplying N by 6.25. Urine samples were analyzed for Kjeldahl N content. Dietary and fecal NDF and ADF were determined on composite samples according to Van Soest et al. [10] using an Ankom 200 fiber analyzer and ANKOM F57 filter bags (Ankom Technology Corp., Fairport, NY).

### Condensed tannin analysis

Aqueous acetone (70 %) extractable CT in the diets were determined using butanol-HCl [11]. Tannin composition of whole PB, aqueous acetone extracts and PB residue after extraction were also analyzed by thiolytic degradation as described by Kommuru et al. [12, 13]; these are described below as total CT, extractable and unextractable CT, respectively.

The following parameters were obtained: mean degree of polymerization (mDP), which describes the average number of flavanol-3-ol monomers per tannin polymer, % prodelphinidin and % procyanidin (% PC or PD) within CT and % *cis* and % *trans*-flavan-3-ols within CT (% cis or trans) plus information on flavanol-3-ols in extender and terminal positions of CT.

### Statistical analysis

Data were analyzed by the Mixed Model procedure of the SAS (SAS, Inst., Inc., Cary, NC) for a completely randomized design with the factors examined being included treatments, periods, and treatment by periods interactions. Linear and quadratic effects were determined utilizing poly-nominal orthogonal contrasts for equally spaced treatments. Animals were the experimental unit and were treated as a random effect. The variables included were diet-composition, feed intake, nutrient digestibility, N-balance, and mineral retention. Mineral retention in the body was calculated from total mineral intake minus fecal mineral composition. Data are presented as least squares (LS) mean values together with the standard deviation (SD) and standard error of the mean (SEM). There was no treatment x period interactions (*P* > 0.10), hence only the main effects are reported for rumen digestibility in the result section.

## Results

### Ingredients and chemical composition of experimental diets and diet ingredients

Ingredients and chemical composition of experimental diets, PB, WS and BGH are presented in Table 1. Total CT concentration in the PB and WS was 10.3 and 0.03 % DM, respectively. However, grain mix analysis resulted in 0.19, 1.63 and 3.2 % CT on % DM for the 0, 15 and 30 % PB diets. All experimental treatments provided similar nutrients, except for CT and lignin that were higher in 15 and 30 % PB ration.

Tannin analysis (Table 2) revealed that epicatechin was the major extended unit in the total CT (67.4 %), extractable CT (38.2 %), and unextractable CT (74.0 %). Catechin was the major terminal unit in the total CT (7.1 %) and extractable CT (8.4 %), but gallocatechin was the major terminal unit (30.4 %) in extractable CT (30.4 %). PB CT were mostly procyanidins (Table 2): total CT consisted of 87.6 % PC and 12.4 % PD, extractable CT of 54.8 % PC and 45.2 % PD and unextractable CT of 94.5 % PC and 5.5 % PD. Extractable CT had oligomers with mDP-values of 2.64 and unextractable CT had polymers with mDP-values of 11.1.

**Table 2:** Condensed tannin and flavonol compositions of pine bark after thiolysis with benzyl mercaptan

Item	Pine bark			
	Total CT	Extractable CT	Unextractable CT	SD
Condensed tannins (CT)				
CT (Bu-HCL)	10.3	8.7	1.7	0.15
CT (Thiolysis)				
mDP	10.5	2.6	11.1	0.15
%PC in CT	87.6	54.8	94.5	0.73
%PD in CT	12.4	45.2	5.5	0.36
% cis-flavan-3-ols in CT	76.9	48.72	80.2	0.46
% trans-flavan-3ols in CT	23.1	51.28	19.8	0.57
Flavan-3-ol composition of CT (%)				
^a^ GC	2.0	30.4	0.0	0.15
^a^ EGC	0.0	0.0	0.0	0.0
^a^ C	7.1	7.6	8.4	0.26
^a^ EC	0.6	0.0	0.7	0.02
GC-BM	1.5	4.3	0.0	0.12
EGC-BM	8.9	10.5	5.5	0.57
C-BM	12.7	9.0	11.4	0.55
EC-BM	67.4	38.2	74.0	0.74

*CT* condensed tannins, *PC* procyanidins, *PD* prodelphinidins, *GC* gallocatechin, *EGC* epigallocatechin, *C* catechin, *EC* epicatechin, *mDP* mean degree of polymerization; flavan-3-ols composition is expressed in terms of relative molar percentages; ^a^: flavan-3-ols in terminal position of the tannins; *BM* benzylmercaptan adduct, for flavan-3-ols in extender position

### In vivo intake and digestibility

Nutrients intake is summarized in Table 3. Nitrogen intake and fecal N excretion were not affected (*P* > 0.05) by addition of PB in the diet (Table 3), but N balance in the body was quadratically increased (*P* < 0.01) in the 15 % PB diet compared to other diets. This may be due to increased rumen escape protein and less excreted urinary N with the 15 % PB diet.

**Table 3:** Nitrogen utilization by goats fed various levels of pine bark (PB) supplementation

	Treatment (% PB)			*P*-value*		
Item	0	15	30	SEM	Linear	Quadratic
No. of animals	6	6	6			
N intake, g/d	24.3	26.1	26.5	2.14	0.48	0.80
Fecal N, g/d	6.2	6.6	7.4	0.67	0.21	0.84
Urinary N, g/d	6.6	6.4	9.1	0.87	0.06	0.18
N-balance, g/d	11.5	13.1	10.0	1.01	0.33	0.01
N-balance, % N intake	47.0	50.0	37.0	3.92	0.28	0.05

* Based on orthogonal contrast for equally spaced treatments

There were no treatment × period interactions (*P* > 0.10) hence only the main effects are reported

Average total dry matter intake (DMI), fecal DM output, nutrients digestibility and major mineral utilization of diets are summarized in Table 4. Average body weight (BW) and NDF intakes were similar among treatments, but ADF (*P* < 0.05), lignin (*P* < 0.01), NFC (*P* < 0.02), CT (*P* < 0.001), and TDN (*P* < 0.06) intakes were linearly increased as PB increased in the diets. In contrast, DM (*P* < 0.03), NDF (*P* < 0.02), and ash (*P* < 0.04) intakes were quadratically decreased with PB supplementation.

**Table 4:** Apparent nutrient intake, digestibility and major mineral utilization of diets by goats consuming various levels of pine bark (PB) supplementation

	Treatment (% PB)				*P*-value*	
Item	0	15	30	SEM	Linear	Quadratic
No. of animals	6	6	6			
Average BW	33.2	31.1	31.8	1.49	0.52	0.47
DMI, kg/d	1.02	1.01	1.10	0.80	0.49	0.68
Digestible DMI, kg/d	0.68	0.62	0.76	0.25	0.25	0.05
Digestible CP intake,	119.4	121.4	106.8	7.50	0.43	0.28
kg/d						
Intake, g/kg BW						
DM	31.3	28.8	34.3	1.36	0.12	0.03
CP	4.7	4.5	5.2	0.23	0.12	0.17
NDF	11.9	10.4	11.1	0.40	0.16	0.02
ADF	7.8	7.1	8.6	0.30	0.05	0.06
Lignin	1.94	3.2	4.0	0.10	0.01	0.07
NFC	12.1	11.1	14.3	0.66	0.02	0.06
Ash	1.96	1.75	1.97	0.08	0.94	0.04
CT (butanol-HCl)	0.06	0.44	1.11	0.02	0.001	0.01
TDN, %	66.6	64.1	64.5	0.78	0.06	0.16
Fecal DM output, g/d	347.8	334.0	373.9	32.56	0.58	0.51
Fecal DM output, g/BW	10.6	10.5	12.0	0.56	0.09	0.28
Digestibility, %						
DM	66.3	63.3	65.0	1.67	0.59	0.26
CP	73.5	71.1	69.6	1.64	0.09	0.84
NDF	48.8	39.4	36.5	2.84	0.01	0.36
ADF	47.4	34.2	29.7	3.45	0.001	0.30
NFC^a^	63.1	58.2	60.1	2.12	0.33	0.19
Lignin	41.1	27.7	18.2	4.50	0.001	0.73
Ash	65.5	62.3	64.3	1.74	0.61	0.23
Digested mineral^b^, g/d						
Ca	37.8	41.5	45.6	3.21	0.09	0.95
P	2.9	9.4	20.7	5.44	0.03	0.72
Mg	46.5	42.8	57.4	2.98	0.01	0.02
K	79.8	71.9	61.2	3.47	0.001	0.75
S	61.9	59.4	56.9	2.19	0.11	0.99
Na	27.0	41.8	33.5	5.03	0.13	0.11
Cu, mg/d	62.8	63.2	47.1	6.02	0.07	0.27
Mn, mg/d	20.3	33.9	36.8	4.35	0.01	0.33
Zn, mg/d	28.7	30.9	46.6	4.37	0.01	0.22
Fe, mg/d	12.6	20.2	21.8	3.68	0.09	0.51

* Based on orthogonal contrast for equally spaced treatments

^a^ *NFC* non-fiber carbohydrate, *DMI* dry matter (DM) intake, *CP* crude protein, *NDF* neutral detergent fiber, *ADF* acid detergent fiber

^b^ Digested mineral = Intake of minerals (g/DM)–fecal mineral contents (g/DM) during 24 h sample collection

Digestibility of DM, NFC and ash were similar among treatment, but digestibility of CP (*P* = 0.09), NDF (*P* < 0.01), ADF (*P* < 0.001) and lignin (*P* < 0.001) declined linearly as PB increased in the diets (Table 4). The lack of a quadratic response in digestibility of CP, NDF, and ADF to dietary concentration of PB suggests that protein and fiber digestibility responses were linear. Increasing PB supplementation did not affect (*P* > 0.10) daily DMI (g/d), daily fecal DM output (g/d), DM digestibility, NFC and ash digestibility. Across the BW (g DM/kg BW), however, DMI per gram of BW (quadratic; *P* < 0.03), digestible DMI (quadratic; *P* < 0.05), and fecal DM output (linear; *P* = 0.09) in the 15 % PB or non-PB supplemented animals were lower than in animals receiving the 30 % PB supplemented group. The quadratic response in DMI per kg BW in the present experiment may be interpreted to indicate that DMI was proportionally more increased when diets contained a higher concentration of dietary PB up to 30 %.

Amount of K digested decreased linearly (*P* < 0.001) as did S (*P* = 0.1) and Cu (*P* = 0.07) with PB supplementation whereas amount of P digested increased linearly (*P* < 0.03), as did Mg (*P* < 0.01), Mn (*P* < 0.01), Zn (*P* < 0.01) and Fe (*P* = 0.09) as PB increased in the diets.

## Discussion

The principal objectives of this study were to measure the effects of CT-containing PB supplementation as a feed replacement on feed intake, ruminal digestibility, nitrogen balance, and mineral retention in meat goats. The most significant findings of this study were increased N-balance and slightly decreased CP digestibility when goats received moderate levels of CT-containing PB (15 %) diet. However, addition of PB up to 30 % to the diets negatively impacted N balance and fiber digestion, likely due to the formation of CT nutrient complexes in the rumen. The linear (*P* < 0.06) and quadrate response (*P* < 0.18) in urinary nitrogen excretion may reflect altered N metabolism in the rumen.

Puchala et al. [14] and Solaiman et al. [15] reported that goats receiving CT-containing forage sericea lespedeza (*Lespedeza cuneata*) based diet (2.2 and 17 % CT DM diets, respectively) had greater DMI than those fed alfalfa hay based diet. Turner et al. [16] suggested that increasing intake with time was a result of rumen adaptation to low quality feed. The quadratic response in DMI per kg BW in the present experiment could be interpreted to indicate that DMI was proportionally increased when diets contained higher concentration of dietary PB up to 30 %.

Waghorn et al. [17] and Min et al. [18] reported that the DMI and DM digestibility in *Lotus corniculatus* (2.2 % CT DM) forage diets were similar between treatment groups, but the apparent digestion of nitrogen was lower in the CT-containing lotus forage diet than in the control (polyethylene glycol treatment; CT-inactive group) sheep. However, animals receiving the high levels of CT-containing big trefoil forage (*Lotus pedunculatus*; 1.4, 4.5 and 9.5 % CT DM) had the apparent digestibility of energy and readily fermentable carbohydrate (soluble carbohydrate + pectin) that decreased linearly with increasing CT content in the diets [2]. Woodward and Reed [19] found that the lower CP digestibility in diets containing the tanninferous *Accacia brevispica* was likely due to tannin-protein complexes [20]. Based on our initial findings animal performance was improved when fed at 15 or 30 % of the diet [6]. Thus, CT in ground PB affected DM intake as well as CP and fiber digestion.

Min et al. [18] reported that the N intake, rumen non-ammonia N pool size, rumen microbial N and abomasal microbial N in *Lotus corniculatus* (2.2 % CT DM) forage diets were similar between treatment groups, but the rumen undegradable protein was greater in the CT-containing lotus forage diet than in the control (polyethylene glycol treatment; CT-inactive group) sheep. Similarly, our study showed that N intake and fecal N excretion were not affected by addition of PB in the diet, but N balance in the body was quadratically increased by the 15 % PB compared to the other diets. This may be due to more rumen undegradable protein and less excreted urinary N in the 15 % PB diet.

The mechanisms by which CT affect mineral retention are poorly understood. For example, it is well known that CT can precipitate proteins, but we do not know whether protein precipitation or metal chelation by the polyphenolic groups CT can affect mineral absorption. The lower linear K (*P* < 0.001), S (*P* = 0.1), and Cu (*P* = 0.07) retention in the PB supplemented group in the present study (Table 4), compared with those receiving the control WS diet, is consistent with another report [17]. This effect may be primarily due to a low apparent absorption pre-abomasum in the CT-containing diet compared with non-CT-containing control diet animal [17]. The CT monomers of catechins (high in green tea) and procyanidins (Pycnogenol extracted from PB, red wine and cranberries) also have effects on plasma antioxidant activity, energy metabolism and vascular system [21]. Lower absorption is probably a consequence of two main factors: 1) complexation between CT and minerals preventing absorption; 2) effects of CT upon the intestinal mucosa resulting in impaired or delayed absorption [22]. However, data in Table 4 show that apparent retention of P (*P* < 0.03), Mg (*P* < 0.01), Mn (*P* < 0.01), Zn (*P* < 0.01) and Fe (*P* = 0.09) increased linearly as PB increased in the diets, and the effects were more pronounced with the 30 % PB diet, and suggest a nutrient-specific effect of CT on minerals in the gastrointestinal track. The presence of CT in the diet has been shown to increase (30 to 93 %) net absorption of essential AA (threonin, valine, isoleucine, leucine, tyrosine, phenylalanine, histidine and lysine) but reduced non-essential AA absorption, compared with control sheep [17]. Similar mechanisms may be involved in the reactions between dietary CT and minerals, with the minerals that are more selectively absorbed offering more opportunity for retention in the body to be improved through the action of CT.

McNabb et al. [20] reported on the digestion of plant proteins in relation to different types of CT from *Lotus corniculatus* (Birdsfoot trefoil; CT that consist largely of procyanidins) and *Lotus pedunculatus* (big trefoil; CT that are largely prodelphinidins). The amount of tannins required to precipitate all the plant proteins when incubated with CT from *L. corniculatus* and *L. pedunculatus* was similar. Although CT from both species were able to reduce in vitro degradation of plant proteins, CT from *L. pedunculatus* were more effective than CT from *L. corniculatus* at reducing protein degradation. Data from thiolysis revealed that epicatechin was the major extension unit in total CT (67.4 %), extractable CT (38.2 %), and unextractable CT (74.0 %), while catechin and gallocatechin were the major terminal units. This means that PB tannins were mostly procyanidins. Our findings indicate that procyanidin content rather than just protein precipitation capacity may be important for determining the ruminal digestibility of protein in livestock [3].

Current research has indicated that CT in PB affect DMI, CP, minerals and fiber digestion, and impacted positively on N balance when animals received a moderate level of PB in their diet. Nonetheless, the mechanism for decreasing or increasing mineral retention in goats is unknown at this time, and the potential value of CT-containing PB associated with mineral partitioning needs to be tested experimentally. Our findings emphasize the need to further test assumption for the biological role of CT-containing PB in ruminants.

## Abbreviations

ADF: Acid detergent fiber
BW: Body weight
CP: Crude protein
CT: Condensed tannins
DM: Dry matter
NDF: Neutral detergent fiber
NFC: Non-fiber carbohydrate
PB: Pine bark
TDN: Total digestible nutrient
WS: Wheat straw
